# The modulating impact of cigarette smoking on brain structure in panic disorder: a voxel-based morphometry study

**DOI:** 10.1093/scan/nsaa103

**Published:** 2020-07-30

**Authors:** Stefanie L Kunas, Kevin Hilbert, Yunbo Yang, Jan Richter, Alfons Hamm, André Wittmann, Andreas Ströhle, Bettina Pfleiderer, Martin J Herrmann, Thomas Lang, Martin Lotze, Jürgen Deckert, Volker Arolt, Hans-Ulrich Wittchen, Benjamin Straube, Tilo Kircher, Alexander L Gerlach, Ulrike Lueken

**Affiliations:** Department of Psychology, Humboldt-Universität zu Berlin, Berlin 10117, Germany; Department of Psychiatry and Psychotherapy, Campus Charité Mitte, Charité - Universitätsmedizin Berlin, Berlin 10117, Germany; Department of Psychology, Humboldt-Universität zu Berlin, Berlin 10117, Germany; Department of Psychiatry and Psychotherapy and Center for Mind Brain and Behavior (CMBB), Philipps-University Marburg, Marburg 35037, Germany; Department of Biological and Clinical Psychology/Psychotherapy, University of Greifswald, Greifswald 17489, Germany; Department of Biological and Clinical Psychology/Psychotherapy, University of Greifswald, Greifswald 17489, Germany; Department of Psychiatry and Psychotherapy, Campus Charité Mitte, Charité - Universitätsmedizin Berlin, Berlin 10117, Germany; Department of Psychiatry and Psychotherapy, Campus Charité Mitte, Charité - Universitätsmedizin Berlin, Berlin 10117, Germany; Department of Clinical Radiology, University of Münster, Münster 48149, Germany; Department of Psychiatry, Psychosomatics and Psychotherapy, Center of Mental Health, University Hospital of Würzburg, University of Würzburg, Würzburg 97080, Germany; Christoph-Dornier-Foundation for Clinical Psychology, Bremen 28359, Germany; Department of Psychiatry and Psychotherapy, University of Hamburg, Hamburg 20146, Germany; Functional Imaging Unit, Institute for Diagnostic Radiology and Neuroradiology, University of Greifswald, Greifswald 17489, Germany; Department of Psychiatry, Psychosomatics and Psychotherapy, Center of Mental Health, University Hospital of Würzburg, University of Würzburg, Würzburg 97080, Germany; Department of Psychiatry, University of Münster, Münster 48149, Germany; Institute of Clinical Psychology and Psychotherapy, Technische Universität Dresden, Dresden 01069, Germany; Department of Psychiatry and Psychotherapy and Center for Mind Brain and Behavior (CMBB), Philipps-University Marburg, Marburg 35037, Germany; Department of Psychiatry and Psychotherapy and Center for Mind Brain and Behavior (CMBB), Philipps-University Marburg, Marburg 35037, Germany; Department of Psychiatry and Psychotherapy and Center for Mind Brain and Behavior (CMBB), Philipps-University Marburg, Marburg 35037, Germany; Department of Psychology, Humboldt-Universität zu Berlin, Berlin 10117, Germany

**Keywords:** smoking, gray matter volume, panic disorder, amygdala, hippocampus

## Abstract

Cigarette smoking increases the likelihood of developing anxiety disorders, among them panic disorder (PD). While brain structures altered by smoking partly overlap with morphological changes identified in PD, the modulating impact of smoking as a potential confounder on structural alterations in PD has not yet been addressed. In total, 143 PD patients (71 smokers) and 178 healthy controls (62 smokers) participated in a multicenter magnetic resonance imaging (MRI) study. T1-weighted images were used to examine brain structural alterations using voxel-based morphometry in a priori defined regions of the defensive system network. PD was associated with gray matter volume reductions in the amygdala and hippocampus. This difference was driven by non-smokers and absent in smoking subjects. Bilateral amygdala volumes were reduced with increasing health burden (neither PD nor smoking > either PD or smoking > both PD and smoking). As smoking can narrow or diminish commonly observed structural abnormalities in PD, the effect of smoking should be considered in MRI studies focusing on patients with pathological forms of fear and anxiety. Future studies are needed to determine if smoking may increase the risk for subsequent psychopathology via brain functional or structural alterations.

## Introduction

Smoking behavior is overrepresented in patients with mental disorders in general (Lasser *et al.*, 2000; Cook *et al.*, 2014) and in patients with anxiety disorders particularly (Johnson *et al.*, 2000). Among anxiety disorders, panic disorder (PD) is linked to cigarette smoking in many epidemiological investigations using cross-sectional designs (Goodwin and Hamilton, 2002; Lawrence *et al.*, 2010). Prospective epidemiological studies support smoking to increase the likelihood of developing panic attacks and PD (Breslau and Klein, 1999; Johnson *et al.*, 2000; Isensee *et al.*, 2003; Breslau *et al.*, 2004). Furthermore, PD patients who smoke report significantly more intense anxiety symptoms and greater severity of panic symptoms than those who do not smoke (Zvolensky *et al.*, 2005). In addition, they show increased problems to stop smoking compared to healthy smokers (Piper *et al.*, 2010). However, while the link between smoking and PD is well established epidemiologically, its neurobiological basis remains mostly unclear.

Persistent smoking has been related to a number of structural brain changes following nicotine consumption, as demonstrated by previous cross-sectional studies (Brody *et al.*, 2004; Gallinat *et al.*, 2006; Zhang *et al.*, 2011; Liao *et al.*, 2012; Pan *et al.*, 2013; Fritz *et al.*, 2014). Voxel-based morphometry (VBM) analyses found reduced gray matter volumes (GMV) in the anterior cingulate cortex (ACC; Brody *et al.*, 2004; Yu *et al.*, 2011; Liao *et al.*, 2012), the dorsolateral prefrontal cortex (Brody *et al.*, 2004; Gallinat *et al.*, 2006; Liao *et al.*, 2012; Fritz *et al.*, 2014), the orbitofrontal cortex (OFC; Kühn *et al.*, 2010; Morales *et al.*, 2012; Fritz *et al.*, 2014), the fusiform gyrus (Gallinat *et al.*, 2006), the cerebellum (Brody *et al.*, 2004; Kühn *et al.*, 2012) and in the left thalamus (Liao *et al.*, 2012; Hanlon *et al.*, 2016). Associations between cigarette smoking and brain volumes were also identified for striatal nuclei, with smaller nucleus accumbens volumes (Das *et al.*, 2012) and greater putamen volumes (Das *et al.*, 2012; Franklin *et al.*, 2014) in otherwise healthy smokers. Contradictory evidence is available for amygdala volume, where Durazzo *et al.* (2017) reported smaller GMV, whereas Shen *et al.* (2017) did not find any differences in healthy smokers *vs* non-smokers.

Neural system models for PD emphasize altered functionality of a network conferring defensive reactivity, which encompasses the insula, ACC, thalamus, hippocampus, amygdala and regions of the brain stem (midbrain, periaqueductal gray; Dresler *et al.*, 2013). Structural alterations have been reported for limbic structures (amygdala, hippocampus), cortical areas (ACC), the brain stem (midbrain, pons), basal ganglia (caudate, putamen) and the thalamus (Massana *et al.*, 2003; Uchida *et al.*, 2003; Asami *et al.*, 2009; Hayano *et al.*, 2009; Del Casale *et al.*, 2013; Dresler *et al.*, 2013). Reduced volumes of cortico-limbic structures were associated with PD symptoms and maintenance (Dresler *et al.*, 2013). It appears that brain structural abnormalities in smokers substantially overlap with those associated with PD pathophysiology in terms of fronto-limbic circuits (e.g. ACC, amygdala). As smoking behavior is overrepresented among PD patients, it may represent a potential confounder. Thus, differences between PD patients and healthy controls (HC) may have been over- or underestimated in previous investigations as a result of smoking. The same may be true for studies comparing PD patients with other patient groups with lower smoking prevalence.

Only a handful of neuroimaging studies have focused on understanding the neural mechanisms of smoking and comorbid mental illness, and the majority of these studies have concentrated on comorbid schizophrenia (Tregellas *et al.*, 2007; Schneider *et al.*, 2014; Jørgensen *et al.*, 2015). No investigation has previously examined the effect of smoking on brain morphology in patients with PD. For those areas, overlapping in both conditions, it is unclear whether smoking enhances or obscures the effect of PD.

To address this issue, we here intended to further clarify the modulating impact of smoking on brain morphological correlates in PD patients free of psychopharmacotherapy. First, we aimed to confirm smoking effects on brain morphology in healthy smokers and non-smokers. Second, based on the above-cited VBM studies, we hypothesized GMV reductions in fronto-limbic circuitry, frequently observed as a feature of PD pathophysiology, may be partly driven by differential rates in smoking behaviors. Third, we examined a possible additive effect of smoking on brain morphology in PD patients.

## Materials and methods

### Participants

The study was part of the German national research network PANIC-NET (second funding period). Magnetic resonance imaging (MRI) measurements were conducted in five German centers (Marburg, Berlin, Dresden, Greifswald and Muenster), which are participating centers for the national research initiative PANIC-NET (funded by the German Federal Ministry of Education and Research, BMBF). These centers have a long-standing tradition of collaborative multicenter functional magnetic resonance imaging (fMRI) studies (e.g. Kircher *et al.*, 2013). The current analysis encompasses a *post-hoc* research question supplementing the main study outcomes. In total, 157 PD patients and 187 HC subjects underwent MRI scanning. Inclusion criteria for patients were as follows: (i) a current DSM-IV-TR (Diagnostic and Statistical Manual of Mental Disorders 4th Edition) primary diagnosis of PD (American Psychiatric Association, 2000) evidenced by the Composite International Diagnostic Interview (CAPI-WHO-CIDI; DIAX-CIDI version) and validated by clinical experts; (ii) a score ≥3 on the Clinical Global Impressions Scale and (iii) an age of 18–65 years. Exclusion criteria were as follows: (i) comorbid DSM-IV-TR psychotic or bipolar I disorder; (ii) current alcohol dependence/current abuse or dependence on benzodiazepine and other psychoactive substances; (iii) current suicidal intent; (iv) borderline personality disorder; (v) concurrent ongoing psychopharmacological treatment for PD or another mental disorder and (vi) antidepressant or anxiolytic pharmacotherapy. The HC group was free of current or past medical, neurological or mental illness as evidenced by a clinical interview. Additional MRI-related exclusion criteria such as ferromagnetic metal implants applied to both groups. Smoking status was assessed on a categorical level (yes/no) by self-report and compared with 12-month DSM-IV-TR diagnosis of nicotine dependence in the PD group, assessed in the clinical interview. After exclusion of participants with missing data regarding smoking status and quality control, MRI data from 143 PD patients [72 non-smokers (PD/NS) and 71 smokers (PD/S)] and 178 HC participants [116 non-smokers (HC/NS) and 62 smokers (HC/S)] were included in the present analysis (see Figure S1 in the supplement). Sociodemographic and clinical characteristics of the final, ‘quality controlled’ sample are shown in Table 1. The study was approved by the ethics committees of all participating universities. All subjects gave written, informed consent before participating in the study.

**Table 1:** Sociodemographic characteristics of the smoker and non-smoker sample and clinical characteristics of the PD patients sample, only

	Smokers, mean +/− s.d. or no. (%) n = 133				Non-smokers, mean +/− s.d. or no. (%) n = 188			
Sociodemographic characteristics								
PD (n = 71) 53%		HC (n = 62) 47%	Statistic *t* or χ^2^	*P*	PD (n = 72) 38%	HC (n = 116) 62%	Statistic *t* or χ^2^	*P*
Age [mean (s.d.)]	34.13 (10.7)	31.23 (9.5)	1.643	0.103	33.18 (11.3)	31.85 (10.8)	0.804	0.423
Female gender [n (%)]	45 (63)	34 (55)	1.001	0.317	44 (61)	67 (58)	0.206	0.650
Years of education [n (%)]								
8	5 (7)	0	10.413	0.005	4 (6)	1 (1)	16.427	<0.001
10	23 (32)	10 (16)			26 (36)	17 (15)		
12–13	43 (61)	52 (84)			42 (58)	97 (84)		
Right-handedness [n (%)]	69 (97)	62 (100)	0.904	0.342	69 (96)	112 (97)	0.850	0.654
Clinical characteristics								
	PD patients, mean +/− s.d. or no. (%) n = 143					HC subjects, mean +/− s.d. or no. (%) n = 178			
	Smokers (n = 71) 50%		Non-smokers (n = 72) 50%	Statistic *t* or χ^2^	*P*	Smokers (n = 62) 35%	Non-smokers (n = 116) 65%	Statistic *t* or χ^2^	*P*
SIGH-A	20.4 (7.6)	18.6 (8.8)	1.276	0.204	2.3 (2.3)	1.8 (2.1)	1.170	0.244
ASI	32.8 (11.5)	30.0 (11.5)	1.449	0.150	10.31 (6.3)	10.0 (6.7)	0.305	0.761
BSI	62.4 (32.9)	51.6 (34.0)	1.900	0.060	6.9 (7.1)	8.0 (9.2)	−0.735	0.453
BDI	14.8 (8.5)	12.3 (8.3)	1.760	0.081	2.2 (2.9)	2.0 (2.6)	0.374	0.709
MI total	2.2 (0.9)	2.1 (0.7)	0.851	0.396				
MI AAC	1.9 (0.9)	1.8 (0.7)	0.653	0.515				
MI AAL	2.5 (0.9)	2.4 (0.9)	0.782	0.436				
CGI	4.5 (1.2)	4.2 (0.9)	1.530	0.128				
PAS	23.2 (8.5)	21.7 (9.4)	0.994	0.322				
Comorbid DEP	32 (45.10)	19 (26.39)	5.437	0.020				
Comorbid diagnoses	1.54 (1.34)	1.14 (1.43)	1.70	0.10				
Nicotine dependence^a^	20 (28.17)	6 (8.33)						

*Notes*: PD, panic disorder; HC, healthy controls; CGI, Clinical Global Impression Scale; SIGH-A, Hamilton Anxiety Scale; PAS, Panic and Agoraphobia Scale; ASI, Anxiety Sensitivity Index; BDI-II, Beck Depression Inventory; BSI, Brief Symptom Inventory; MI total, Mobility Inventory total score; MI AAC, Mobility Inventory Avoidance Accompanied; MI AAL, Mobility Inventory Avoidance alone; DEP, depressive disorders.

Missing values in HC subjects: SIGH-A: 15 S, 33 NS; ASI: 11 S, 6 NS; BSI: 11 S, 7 NS.

^a^The diagnosis of nicotine dependence is based on the 12-month prevalence assessed by the CIDI interview, the six subjects in the non-smoker group can be characterized as ex-smoker.

### MRI acquisition

MRI data were acquired using 3T scanners. The following scanners were used: a 3T Philips Achieva scanner (Philips Medical Systems, Best, The Netherlands) in Münster; a 3T Siemens Trio scanner (Siemens AG, Erlangen, Germany) in Dresden and Marburg; a 3T Siemens Verio scanner (Siemens AG, Erlangen, Germany) in Greifswald; a 3T General Electric Healthcare scanner (General Electric Healthcare, Milwaukee, WI) and a 3T Siemens Trio scanner in Berlin. MP-RAGE T1-weighted images were acquired with the following parameters: voxel size = 1 × 1 × 1 mm^3^; repetition time (TR) = 1900 ms; inversion time (TI) = 900 ms; field of view (FOV) = 256 × 256 mm^2^; slices per slab = 176; thickness = 1 mm; flip angle = 9, echo time (TE) = 2.26 ms.

### Preprocessing and statistical analyses

We used SPM12 (www.fil.ion.ucl.ac.uk) and the CAT12 toolbox (http://www.neuro.uni-jena.de/cat/) implemented in MATLAB R2016a (MathWorks, Sherborn, MA) to pre-process and analyze the neuroimaging data. Brain scans were segmented in gray matter, white matter and cerebrospinal fluid and subsequently normalized to the Montreal Neurological Institute reference brain in CAT12. The voxel size was re-sampled to 1.5 × 1.5 × 1.5 mm^3^ during this step. The resulting images were quality controlled via visual inspection and CAT12-based outlier checks (homogeneity analysis). All scans that were scored as having low quality in one of the assessments were rejected, which lead to the exclusion of *n* = 16 subjects. Scans from the remaining subjects were smoothed with an 8 mm full-width at half-maximum Gaussian kernel. To confirm pervious findings, we investigated the effect of smoking in HC subjects on a whole-brain level ‘as well as in specific regions pertaining to the pathophysiology of smoking and PD’ (HC/S < HC/NS). Subsequently, we examined our a priori defined hypothesis if actual smoking status confounds differences in specific fronto-limbic circuitry and on a whole-brain level between HC subjects and PD patients by conducting *t*-tests for the smoker and non-smoker groups separately [PD < HC (non-smokers only); PD < HC (smokers only)]. In addition, we examined in an explorative manner the main and interaction effects of smoking and diagnosis [(PD < HC (whole sample); S < NS (whole sample); S < NS (PD only); PD < HC (non-smokers < smokers)]. Furthermore, an explorative analysis on white matter volume differences in pre-defined regions of interest (ROIs) was performed using the above-mentioned contrasts. Age, gender, education, total intracranial volume, study center and Beck Depression Inventory (BDI-II; Beck *et al.*, 1996) scores were included as covariates of no interest. For all analyses, an implicit mask with an absolute threshold of 0.15 was applied, allowing us to include only those voxel showing an increased probability to contain the analyzed tissue type. An anatomical ROI of the a priori defined brain areas (amygdala, ACC, hippocampus, insula, thalamus, OFC) was calculated combining the definitions from the Automated Anatomical Labeling Atlas (Tzourio-Mazoyer *et al.*, 2002) as implemented in the Wake Forest University PickAtlas (Maldjian *et al.*, 2003) in SPM12 in one mask. Small volume correction on this ROI masks was applied using a cluster-forming threshold of *P* < 0.001 on the voxel level and a clusterwise familywise error-corrected threshold of *P*_fwe_ < 0.05 with a minimum cluster size of *k* = 10 contiguous voxels. For the exploratory whole-brain analysis, as recommended in cluster-extent-based thresholding in fMRI analysis (Woo *et al.*, 2014), a cluster-extent threshold was applied to correct for multiple comparisons using *P* < 0.001 as significance threshold on the voxel level and *k* = 200 contiguous voxels on the cluster level. In addition, we performed Pearson’s correlation analyses between significant reduced GMV in PD patients (PD < HC) identified in our ROI analysis and clinical scores (Hamilton Anxiety Scale and Panic and Agoraphobia Scale). To further examine an additive effect of smoking and PD, we conducted a linear regression model in SPM using three groups with increasing health burden (group 1: neither PD nor smoking; group 2: either PD or smoking; group 3: both PD and smoking) as independent variable. We examined a positive and negative linear effect on the previous identified brain regions of the group analysis only.

## Results

### Sample characteristics

Smokers were significantly more frequent in PD patients than in HC subjects [χ^2^ (1) = 7.176, *P* = 0.007]. Demographic data and clinical characteristics are shown in Table 1, where differences within the smoker and non-smoker groups (PD/S *vs* HC/S and PD/NS *vs* HC/NS) are reported. Age, gender and handedness were matched between the two diagnostic groups (PD *vs* HC) and did not significantly differ. However, there was a difference regarding the years of education, with HC subjects showing more years of education than PD patients irrespective of their smoking status. For that, education was added as covariate of no interest in the models. Regarding the clinical characteristics of the PD patient sample only (PD/S *vs* PD/NS), PD/S showed trend wise higher Brief Symptom Inventory and BDI-II scores, reflecting the previously reported association of smoking with symptom severity (Zvolensky *et al.*, 2005). Furthermore, significantly more PD/S suffered from a comorbid depressive disorder ( Table 1), for that, we included the BDI-II scores as covariate of no interest in the models. Based on the 12-month prevalence, assessed by the clinical interview, 20 PD/S patients were characterized as nicotine dependent according to DSM-IV criteria and six PD/NS patients presented nicotine dependence within the past 12 months and can be characterized as ex-smoker.

### VBM: ROI analysis

**Fig. 1:**
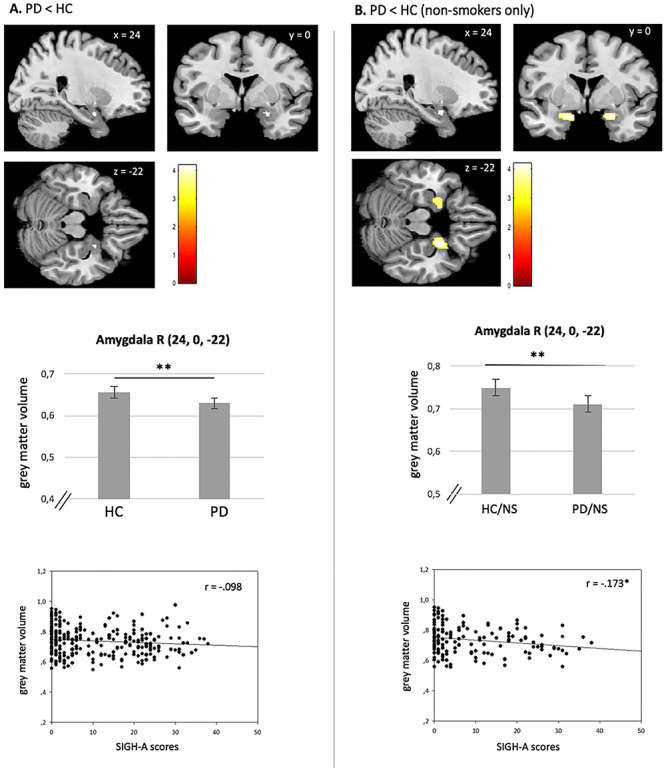
(A): Main effect of PD on GMV across the whole sample of PD patients and HC subjects. PD patients show reduced volumes compared to HC participants in the right amygdala. ‘No significant correlation between GMV and SIGH-A scores’. (B): Significant differences between HC/NS and PD/NS in the right amygdala. Reduced GMVs are negatively correlated with SIGH-A scores. Bars represent the estimated gray matter volumes of the corresponding brain region; error bars represent SEM. R = right. ^*^P < 0.05; ^**^P < 0.001.

Compared to non-smokers, we identified significant regional GMV reductions in smokers in the right insula cortex and ACC in HC subjects (Table 2). Investigating the modulating impact of smoking on PD pathophysiology, only in non-smokers, PD patients showed significantly reduced regional GMV in the bilateral amygdalae and hippocampi compared to non-smoking HC subjects. In the smoker group, no main effect of diagnosis could be shown (Figure 1 and Table 2).

**Table 2:** Locations of significant gray matter volume differences identified in the ROI analysis in PD and HC subjects as a function of smoking status on peak level with MNI coordinates of local maxima

Contrast/region	Side	Cluster size in voxel	*x*	*y*	*z*	*t*-value	*P* FWE corrected
S < NS (healthy controls only)							
Insula	R	60	42	0	8	3.95	0.002
ACC	R	16	14	47	12	3.05	0.034
S > NS (healthy controls only)				No differential effect			
PD < HC (non-smokers only)							
Amygdala	L	92	−18	0	−22	3.71	0.003
Amygdala	R	101	24	0	−22	3.69	0.003
Hippocampus	L	10	−18	−4	−22	3.34	0.038
Hippocampus	R	30	22	−2	−22	3.70	0.013
PD > HC (non-smokers only)					No differential effect		
PD < HC (smokers only)					No differential effect		
PD > HC (smokers only)					No differential effect		
PD < HC (whole sample)							
Amygdala	R	25	24	0	−22	3.36	0.022
PD > HC (whole sample)					No differential effect		
S < NS (whole sample)							
Orbitofrontal gyrus	L	134	−6	42	−12	3.84	0.001
Insula	R	27	44	−2	4	3.37	0.049
ACC	L	20	−6	45	−4	3.28	0.050
S > NS (whole sample)					No differential effect		
S < NS (PD only)							
Thalamus	L	50	−4	−8	14	3.54	0.001
S > NS (PD only)					No differential effect		
Interaction of smoking and diagnosis					No differential effect		

*Notes*: PD, panic disorder; HC, healthy control; ROI, region of interest; NS, non-smoker; S, smoker; L, left; R, right; *x*, *y*, *z*, MNI coordinates; MNI, Montreal Neurological Institute; ACC, anterior cingulate cortex; FWE, familywise error.

*P* < 0.05 FWE corrected with a minimum cluster size of 10 voxel.

Irrespective of smoking status, a GMV reduction in the right amygdala was identified in the PD group compared to HC subjects in our explorative conducted ROI analysis (Figure 1 and Table 2). Furthermore, we found significant smaller regional GMV in smokers compared to non-smokers regardless of diagnosis in the left orbitofrontal gyrus, the right insular cortex and the left ACC. Regarding PD patients, a significant GMV reduction could be obtained in the left thalamus in smokers compared to non-smokers. No significant effects could be obtained in the exploratory conducted interaction analysis (Table 2).

However, we found a significant negative correlation between Structured Interview Guide for the Hamilton Anxiety Scale (SIGH-A) scores and the right amygdala volume (*r* = −0.173, *P* = 0.040) only in the group of non-smokers [PD < HC (non-smokers only)]. When smokers of both groups were included in the analysis, the correlation was not significant (*r* = −0.098, *P* = 0.107). No other significant correlations could be obtained between the right amygdala and the other clinical scores (Figure 1 and Table 3).

**Table 3:** Pearson’s correlation analyses between significant reduced GMV in PD patients compared to HC subjects identified in the ROI analysis with clinical scores

	SIGH-A (*p*)	PAS (*p*)
Amygdala R (non-smoker)	−0.173 (0.040)^*^	−0.076 (0.295)
Amygdala R (whole group)	−0.098 (0.107)	−0.076 (0.295)

*Notes*: GMV, gray matter volumes; PD, panic disorder; HC, healthy control; ROI, region of interest; SIGH-A, Hamilton Anxiety Scale; PAS, Panic and Agoraphobia Scale; R, right.

Missing values in HC subjects: SIGH-A: 48; PAS: 31.

Analyses of white matter volume revealed no significant alterations in smokers compared to non-smokers and in PD patients compared to HC subjects in the a priori defined ROIs.

### VBM: explorative whole-brain analysis

In the HC group, significant regional GMV reductions were found in smokers compared to non-smokers in the right fusiform gyrus, the right precentral gyrus, the left precentral gyrus and the right supplementary motor area (Table 4).

**Table 4:** Locations of significant gray matter volume differences identified in the whole-brain analysis in PD and HC as a function of smoking status on peak level with MNI coordinates of local maxima

Contrast/region	Side	Cluster size in voxel	*x*	*y*	*z*	*t*-value	*P* uncorrected
S < NS (healthy controls only)							
Fusiform gyrus	R	847	28	−42	−18	4.33	<0.001
Precentral gyrus	L	761	−24	−21	69	4.20	<0.001
Precentral gyrus	R	521	46	−15	56	4.17	<0.001
Supplementary motor area	R	222	2	−2	48	3.69	<0.001
S > NS (healthy controls only)						No differential effect	
PD < HC (non-smokers only)							
Parahippocampal area	R	552	22	0	−22	3.76	0.001
Parahippocampal area	L	210	−18	0	−22	3.76	<0.001
Cerebellum	R	409	32	−75	−50	3.57	<0.001
PD > HC (non-smokers only)					No differential effect		
PD < HC (smokers only)					No differential effect		
PD > HC (smokers only)					No differential effect		
PD < HC							
Middle temporal gyrus	L	267	−40	6	−27	3.97	<0.001
PD > HC					No differential effect		
S < NS (whole sample)							
Fusiform gyrus	R	1463	28	−45	−16	5.20	<0.001
Precentral gyrus	L	337	−36	−16	60	3.71	<0.001
Precentral gyrus	R	325	38	−20	42	3.99	<0.001
Orbitofrontal gyrus inclusive							
ACC	L	208	−6	42	−12	3.84	<0.001
Middle frontal gyrus	L	219	−21	21	48	3.72	<0.001
S > NS (whole sample)				No differential effect			
S < NS (PD only)				No differential effect			
S > NS (PD only)				No differential effect			
Interaction of smoking and diagnosis					No differential effect		

*Notes*: PD, panic disorder; HC, healthy control; NS, non-smoker; S, smoker; L, left; R, right; *x*, *y*, *z*, MNI coordinates; MNI, Montreal Neurological Institute; ACC, anterior cingulate cortex.

*P* < 0.001 uncorrected with a minimum cluster size of 200 voxels.

Related to HC/NS subjects, PD/NS patients showed significantly reduced regional GMV in the left and right parahippocampal area and the right cerebellum. No significant differences in GMV could be obtained for PD/NS > HC/NS. In the smoker group only, no main effect of diagnosis could be shown (Table 4).

The exploratory conducted main effect of diagnosis, irrespective of smoking status, showed a significant reduced GMV in PD patients in the left middle temporal gyrus (Table 4). Irrespective of diagnosis, we found significant smaller regional GMV in smokers compared to non-smokers in the right fusiform gyrus, the right and left precentral gyrus, the left orbitofrontal gyrus inclusive ACC and in the left middle frontal gyrus (Table 4). No additional structural alterations were identified in PD/S patients compared to PD/NS patients on a whole-brain level. The interaction analysis of smoking and diagnosis revealed no significant results.

### Regression analyses

**Fig. 2:**
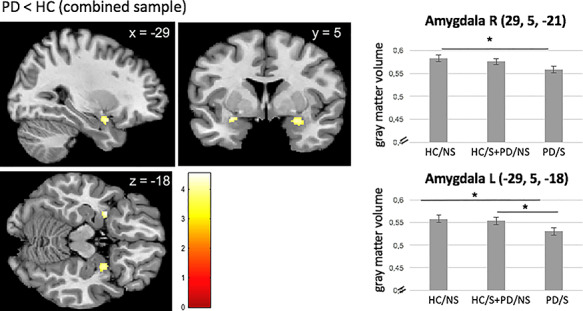
Linear negative association between health burden and bilateral amygdalae GMV. Bars represent the estimated gray matter volumes of the corresponding brain region; error bars represent SEM. R = right, L = left. ^*^P < 0.05.

We performed a regression analysis to examine a potential additive effect of health burden on the previous identified brain regions. We found a linear decrease in the left and right amygdala with increasing health burden in the ROI analysis (Figure 2 and Table 5 as well as Figure S2 in the supplement).

Results did not change when excluding the six ex-smokers from the group of PD/NS.

## Discussion

As supported by the present findings, smoking behavior is highly prevalent in PD patients. Structural abnormalities characterizing the neurobiology of smoking do, to some extent, overlap with brain circuits involved in the pathophysiology of PD. Despite this high co-occurrence, the modulating impact of smoking on regional gray matter abnormalities in PD patients was not explicitly targeted before. Present findings partly confirm pervious identified morphological differences between healthy smokers and non-smokers and indicate that GMV reductions in the amygdala and hippocampus commonly associated with PD pathophysiology are mainly driven by non-smokers. These effects appear to diminish in smokers, which can be attributed to already reduced GMV in healthy smoking subjects. Furthermore, bilateral amygdala volumes show a linear decrease with increasing health burden, possibly indicating additive effects of smoking and PD.

Regarding PD patients, GMV reductions in the bilateral amygdalae and hippocampi were more pronounced in the non-smoker group only than in the combined sample. Furthermore, comparing the smoker group of PD patients and HC subjects yielded comparable GMV in the above-mentioned brain structures. Results of the correlation analysis support this finding. We found a non-significant relationship between amygdala volume and SIGH-A scores, when smokers of both groups were included in the analysis and a significant negative relation in non-smokers only. GMV reductions in the amygdala and hippocampus can be related to the pathophysiology of PD, evidenced by previous research (Del Casale *et al.*, 2013; Pannekoek *et al.*, 2013). These findings on a morphological level can be matched by the presence of neurochemical alterations. PD patients demonstrate lower N-acetylaspartate in the hippocampus (Trzesniak *et al.*, 2010), and reduced binding properties for the serotonin 5-HT_1A_ receptor are evident in the amygdala and hippocampus in PD patients (Nash *et al.*, 2008). Considering the results of the whole-brain approach, the parahippocampal gyrus, a brain region linked to the pathophysiology of PD in several previous studies (Del Casale *et al.*, 2013; Dresler *et al.*, 2013), was significant only when smokers of both groups were excluded from the analysis. Findings indicate that current smoking has indeed a modulating impact on brain morphological correlates in PD patients potentially blurring group differences associated with psychopathology. We thus conclude that current smoking behavior in PD patients and HC subjects can narrow or diminish commonly observed structural abnormalities in PD patients. If this confounder is not considered by matching or as a covariate, which was the case in previous examinations (i.e. Massana *et al.*, 2003; Asami *et al.*, 2009, 2018; Hayano *et al.*, 2009; Uchida *et al.*, 2003), findings on structural abnormalities in PD patients pertaining to key regions involved (e.g. amygdala and hippocampus) may be biased. As such, it can be speculated if previous studies including both smoking and non-smoking subjects may even have underestimated the true effect of volume reduction in the amygdala and hippocampus.

Across the whole sample, PD patients exhibited reduced GMV in a subcortical brain structure compared to HC participants. Studies with different functional and structural imaging modalities have consistently reported abnormalities of the amygdala in PD patients, consistent with animal work on fear conditioning and prominent neuroanatomical models of PD (Gorman *et al.*, 2000; Pannekoek *et al.*, 2013). Reduced amygdala volumes in PD patients may represent the structural basis of the functional abnormalities that have been reported in the neuroimaging literature (Sakai *et al.*, 2005; Pillay *et al.*, 2006; Nash *et al.*, 2008; Chechko *et al.*, 2009; Tuescher *et al.*, 2011).

To further examine if smoking results in an additive effect on the previously identified brain structures, we conducted a regression model. Considering three groups with increasing health burden, a linear negative effect of decreasing GMV with increasing burden was observed for bilateral amygdalae volumes, suggesting that the combined impact of health burden (smoking and PD) may be reflected in the structure of the amygdalae. Previous research linked structural alterations of this brain region to both conditions (Dresler *et al.*, 2013; Durazzo *et al.*, 2017). However, alternatively it needs to be considered that the observed GMV reductions in the amygdalae may have existed even before subjects began smoking and developing panic symptoms and PD. Reduced GMV in the amygdalae could therefore also represent a potentially predisposing factor to smoking behavior or PD, or a shared factor for the development of both conditions.

Considering the HC group, GMV reductions in smokers with respect to *the* ‘insula, ACC’ and fusiform gyrus are consistent with results from previous VBM investigations (Brody *et al.*, 2004; Gallinat *et al.*, 2006; Yu *et al.*, 2011; Liao *et al.*, 2012; Stoeckel *et al.*, 2016). When evaluating the whole sample, smoker showed significant GMV reductions in ‘prefrontal cortex’ regions identified in various studies before, like in the OFC and middle frontal gyrus (Brody *et al.*, 2004; Morales *et al.*, 2012; Fritz *et al.*, 2014), which have been related to the neurobiology of substance addiction, including smoking (Goldstein and Volkow, 2002).

**Table 5:** Regression analysis of the whole sample, separated in three groups with increasing burden of health (group 1: neither PD nor smoking; group 2: either PD or smoking; group 3: both PD and smoking)

	Side	Cluster size in voxel	*x*	*y*	*z*	*t*-value	*P* FWE corrected
Linear negative effect							
Amygdala	L	22	−29	5	−18	3.80	0.017
Amygdala	R	57	29	5	−21	4.4	0.010
Linear positive effect					No differential effect		

*Notes*: PD, panic disorder; L, left; R, right; *x*, *y*, *z*, MNI coordinates; MNI, Montreal Neurological Institute; FWE, familywise error.

Cluster represents region of interest analysis with a cluster-based threshold of *P* < 0.05 FWE corrected and with a minimum cluster size of 10 voxel.

Cigarette smoke components, including nicotine and free radicals, facilitate negative effects on various neurotransmitter systems, neurobiology, the respiratory system and normal neurodevelopmental processes (Niedermaier *et al.*, 1993; Zvolensky *et al.*, 2003; Dwyer *et al.*, 2008; Moylan *et al.*, 2013). Evidence into the pathogenesis of PD supports a role of these developments (Moylan *et al.*, 2013). The importance of specific neurotransmitter systems has been extensively demonstrated in anxiety disorders, with current first-line pharmacological therapies interacting predominantly with the serotonergic, noradrenergic, cannabinoid, cholinergic and dopaminergic systems (Moylan *et al.*, 2013). However, some of these agents are also effective in enhancing smoking cessation (Jorenby *et al.*, 1999), suggesting a plausible biological interaction between these systems and nicotine dependence. Furthermore, free radicals, another highly concentrated component of cigarette smoke, were linked to a relative deficit in both, tryptophan and serotonin, which could be related to increased anxiety symptoms (Bell *et al.*, 2005; Kulz *et al.*, 2007). Numerous population-based studies demonstrated smoking as being prospectively associated with increased rates of anxiety disorders and PD (Breslau and Klein, 1999; Johnson *et al.*, 2000). The effects caused by cigarette smoking may at least partially underpin the biological mechanisms through which smoking might contribute to the development of PD. Hence, it can be speculated that smoking may act as a brain structural vulnerability factor. Like on neurotransmitter systems, smoking cigarettes impacts on specific brain structures also involved in the pathogenesis of PD and thereby possibly predisposing smokers for the development of anxiety symptoms and PD.

Conversely, nicotine can exert an anxiolytic effect as well through rapid desensitization of nicotine acetylcholine receptors in the brain (Gentry and Lukas, 2002; Picciotto *et al.*, 2008) and thereby reduce anxiety symptoms, known as the theory of self-medication or self-treatment. Evidence supports that nicotine exposure does produce a subjective calming effect, although this is coupled with an increase in objective measures of physiological arousal (Perkins, 1995), which could contribute to the maintenance or an increased frequency of smoking in PD patients.

However, as our study is limited by its cross-sectional design, longitudinal studies are clearly needed to assess the role of smoking as a potential marker of vulnerability for PD on a brain structural level.

Although our study benefits from a large sample size, we have to consider some limitations. The study was not primarily designed to examine the effect of smoking, so we had only limited data available on smoking behavior; though a more detailed assessment of smoking severity, history and dependence could shed more light on the observed differences. Furthermore, smoking status was based exclusively on self-report and was not verified by parameters like CO levels or plasma cotinine. As all patients were medication-free, the interaction of cigarette smoking and selective serotonin re-uptake inhibitor (SSRI) treatment could not be investigated. Smokers differ from non-smokers in pharmacokinetics (Zevin and Benowitz, 1999; Kroon, 2007), thus it is plausible to assume an additional interaction with SSRI treatment on symptomatology and possibly brain structure and function. As SSRIs are a first-line treatment, studying the interaction effects with smoking in PD patients would be of relevance from an ecological validity perspective. Significantly more PD/S patients suffered from a comorbid depressive disorder, which seems plausible, considering the link between smoking and symptom severity (Covey *et al.*, 1998; Zvolensky *et al.*, 2005), although our effects remained stable after controlling for depressive symptom scores (BDI-II). Because of the cross-sectional design of our study, we cannot exclude the possibility of any pre-existing structural differences between the analyzed smokers and non-smokers as well as between HC participants and PD patients. As our study represents a preliminary investigation of the modulating impact of actual smoking behavior on commonly established brain structures in PD patients, future studies are encouraged to investigate this interaction in more depth, including elaborate measures on smoking behavior.

We conclude that current cigarette smoking impacts on neural pathways associated with PD pathophysiology in smokers, differences in GMV reductions in the amygdala and hippocampus diminished. In line, we could demonstrate an additive effect of smoking and PD on amygdalae volumes, a brain structure involved in both, the pathophysiology of PD and nicotine dependence. From a methodological perspective, current smoking status should be considered as an important covariate in future neuroimaging studies focusing on PD. From a mechanism-based perspective, the frequently observed co-occurrence of smoking and PD may be reflected by partly overlapping neurostructural correlates. Longitudinal studies are needed to assess whether smoking (which onset precedes the one of PD) may confer its risk properties also via the neural systems level. Early preventive approaches on smoking cessation particularly in PD vulnerable individuals may specifically ameliorate the adverse effects of smoking also on a neural systems level.

### Funding/support and acknowledgments

We acknowledge support by the German Research Foundation (DFG) and the Open Access Publication Fund of Humboldt-Universität zu Berlin.

This work is part of the German multicenter trial: Mechanisms of CBT-treatment effects in patients with panic disorder and panic disorder with agoraphobia: The role of interoceptive exposure and fear augmentation (MCBT-PDAS). The study is funded by the German Federal Ministry of Education and Research (BMBF, 01GV0611) as part of the larger BMBF Psychotherapy Research Funding Initiative Improving the Treatment of Panic Disorder.

Principal investigators (PI) with respective areas of concentration of the MCBT-PDAS are A.H. (Greifswald: PI Psychophysiology); T.L. [Bremen: Study Director for the Randomized Clinical Trial (RCT) and Manual Development]; A.L.G. (Münster: PI Panic Subtypes); Georg W. Alpers (Mannheim: PI Ambulatory Assessment); Christiane Pané-Farré (Greifswald: PI Psychophysiology and Panic Disorder); T.K. (Marburg: PI for Functional Neuroimaging) and J.D. (Würzburg: PI for Genetics). Additional site directors in the RCT component of the program are Winfried Rief (Marburg) and Paul Pauli (Würzburg).

Centers of the Research Network: V.A. (Münster: Overall Network Coordination), H.-U.W. (Dresden) and A.S. (Berlin).

Data Access and Responsibility: All PI take responsibility for the integrity of the respective study data and their components. All authors and co-authors had full access to all study data. Data analysis and manuscript preparation were completed by the authors and co-authors of this article, who take responsibility for its accuracy and content.

Acknowledgments and staff members by site: Bremen (coordinating center for the multicenter trial): Veronika Bamann, Sandra Cammin, Sarah Czilwik, Kira Geisler, Sylvia Helbig-Lang, Kirsten Helmes, Anne Kordt, Tanja Leonhard, Mila Plett-Perelshteyn, Christian Soltau, Juliane Sülz, Maxie von Auer; Dresden: H.-U.W., Nina I. Kleint, U.L.; Greifswald (coordinating site for psychophysiology): Anett Hoffmann, J.R.; Mannheim (coordinating center for ambulatory assessment): Christoph Biwer, Elisabeth Borgmann, Antje Gerdes, Otto Martin, Kristina Steinbach, Bettina Stemmler, Andrew White; Marburg (coordinating center for functional neuroimaging): Tobias Fehlinger, Andreas Jansen, Nikita Jegan, Carsten Konrad, Marion Mickeler, Silke Rusch, Katrin Schlötterer, B.S., Mareike Stumpenhorst, Katrin Wambach, Y.Y.; Münster (coordinating site for panic subtypes): Susanne Kettler, Anna Vossbeck-Elsebusch; Würzburg Psychiatry Department (coordinating center for genetics): Carola Gagel, Andreas Reif, Heike Weber; Würzburg Psychology Department: Almut Friedl-Huber, Harald Krebs, Caroline Ott, Nina Steinhäuser; additional support was provided by the coordinating center for clinical studies in Dresden (KKS Dresden): Marko Käppler. The authors would like to thank Katharina Holtz, Özkan Genc, Johanna Gechter, Kirité Rugani and Anja Balser for their assistance with fMRI data collection.

The study was registered with the NCT01323556.

This neuroimaging study was approved by the Ethics Committee of the Medical Faculty of the Philipps-University Marburg, Germany (Project no. 171/09) and at all local sites.

## Conflict of interest

The following authors report no conflicts of interest concerning the content of this paper: S.L.K., K.H., Y.Y., B.S., U.L., J.R., T.L., B.P., M.L., A.H. and J.D. V.A. is a member of advisory boards and/or gave presentations for the following companies: AstraZeneca, Janssen–Organon, Lilly, Lundbeck, Servier, Pfizer and Wyeth. He has also received research grants from AstraZeneca, Lundbeck and Servier. He chaired the committee for the Wyeth Research Award ‘Depression and Anxiety’. T.K. has received fees for educational programs from Janssen-Cilag, Eli Lilly, Servier, Lundbeck, Bristol Myers Squibb, Pfizer and AstraZeneca; travel support/sponsorship for congresses from Servier; speaker honoraria from Janssen-Cilag; and research grants from Pfizer and Lundbeck. C.K. received fees for an educational program from Aristo Pharma, Janssen-Cilag, Lilly, MagVenture, Servier and Trommsdorff, as well as travel support and speakers honoraria from Aristo Pharma, Janssen-Cilag, Lundbeck, Neuraxpharm and Servier. A.S. has received research funding from the BMBF, the European Commission (FP6) and Lundbeck, and speaker honoraria from AstraZeneca, Boehringer Ingelheim, Lilly, Lundbeck, Pfizer, Wyeth and UCB. Educational grants were awarded by the Stifterverband für die Deutsche Wissenschaft, the Berlin-Brandenburgische Akademie der Wissenschaften, the Boehringer Ingelheim Fonds and the Eli Lilly International Foundation. H.-U.W. has been a member of the advisory boards of several pharmaceutical companies. He has received travel reimbursements and research grant support from Essex Pharma, Sanofi, Pfizer, Organon, Servier, Novartis, Lundbeck and GlaxoSmithKline.

## Supplementary data

Supplementary data are available at *SCAN* online.

## Supplementary Material

nsaa103_Supp
