# Supplementary material for: The modulating impact of cigarette smoking on brain structure in panic disorder: a voxel-based morphometry study
Source: Soc Cogn Affect Neurosci. 2020 Jul 30;15(8):849–59. doi: 10.1093/scan/nsaa103 (PMC7543937; doi:10.1093/scan/nsaa103)
Supplement: nsaa103_Supp [file nsaa103_supp.zip › nsaa103_Supp/scan-19-154-File005.docx]

| 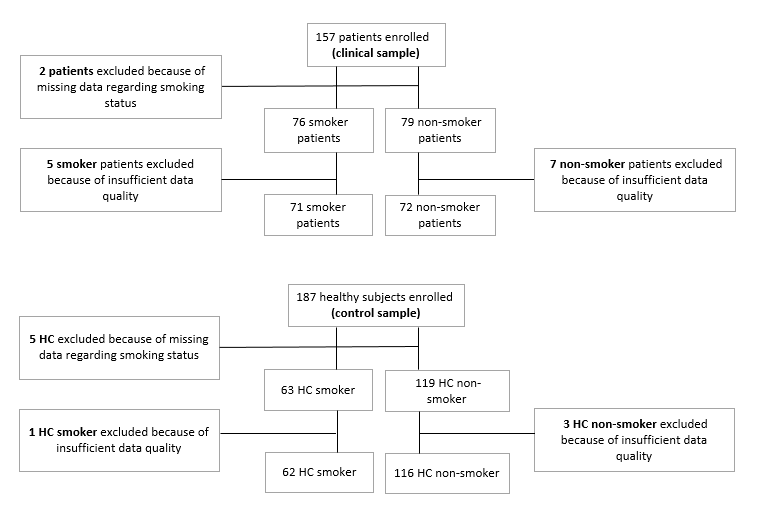 |
| --- |
| **Figure S1:** Flowchart of the sample, separated for PD patients and HC subjects. |

| 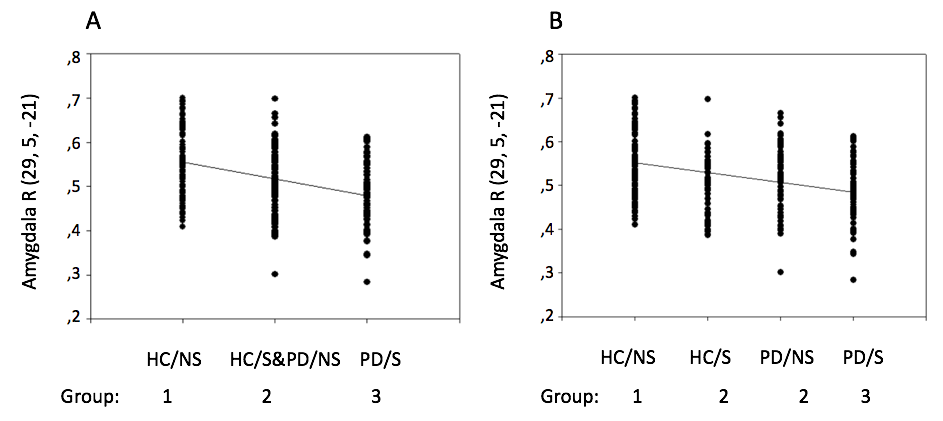 |
| --- |
| **Figure S2**: Scatterplot of right amygdala volumes (extracted from a sphere with 5mm diameter around the peak identified in the regression analysis). Plot A pictures the three groups of the regression analysis and plot B separates group 2 in HC/S & PD/NS. |
